# Improved Prefrontal Activity and Chewing Performance as Function of Wearing Denture in Partially Edentulous Elderly Individuals: Functional Near-Infrared Spectroscopy Study

**DOI:** 10.1371/journal.pone.0158070

**Published:** 2016-06-30

**Authors:** Kazunobu Kamiya, Noriyuki Narita, Sunao Iwaki

**Affiliations:** 1 Department of Removable Prosthodontics, Nihon University School of Dentistry at Matsudo, Chiba, Japan; 2 Automotive Human Factors Research Center, National Institute of Advanced Industrial Science and Technology (AIST), Tsukuba Central 6, Ibaraki, Japan; University of North Carolina at Chapel Hill, UNITED STATES

## Abstract

The purpose of this study was to elucidate the effects of wearing a denture on prefrontal activity during chewing performance. We specifically examined that activity in 12 elderly edentulous subjects [63.1±6.1 years old (mean ± SD)] and 12 young healthy controls (22.1±2.3 years old) using functional near-infrared spectroscopy (fNIRS) in order to evaluate the quality of prefrontal functionality during chewing performance under the conditions of wearing a denture and tooth loss, and then compared the findings with those of young healthy controls. fNIRS and electromyography were used simultaneously to detect prefrontal and masticatory muscle activities during chewing, while occlusal force and masticatory score were also examined by use of a food intake questionnaire. A significant increase in prefrontal activity was observed during chewing while wearing a denture, which was accompanied by increased masticatory muscle activity, occlusal force, and masticatory score, as compared with the tooth loss condition. Prefrontal activation during chewing while wearing a denture in the elderly subjects was not much different from that in the young controls. In contrast, tooth loss in the elderly group resulted in marked prefrontal deactivation, accompanied by decreased masticatory muscle activity, occlusal force, and masticatory score, as compared with the young controls. We concluded that intrinsic prefrontal activation during chewing with a denture may prevent prefrontal depression induced by tooth loss in elderly edentulous patients.

## Introduction

Tooth loss may induce neurodegenerative cognitive decline [1, 2], which has been speculated to be a major risk factor for cognitive impairment [3–7], dementia [4, 8, 9], and Alzheimer’s disease [9, 10], while chewing disability has also been reported to have effects on cognitive decline [11–16], activities of daily living [17, 18], quality of life [19–22], physical functions [11, 12], and mortality rate [23–25]. On the other hand, oral reconstruction using a denture prosthesis may prevent cognitive decline due to tooth loss [26, 27], as use of a denture positively activates chewing-related cortices for prefrontal and parietal sensorimotor cognitive control, as well as parietal and temporal sensory associations [28–31]. However, no known human study has presented neurological evidence of the relationship between chewing deficit caused by tooth loss and cognitive disability, or between chewing activity when wearing a denture and cognitive ability.

Previous investigations have found that wearing a denture activates the prefrontal cortex while chewing as compared to a tooth loss condition, which is accompanied by improved masticatory muscle activity and occlusal contact status in partially edentulous individuals [28, 32]. The prefrontal cortex is involved in both cognitive function and regulation of behavior [33–35]. Therefore, we considered that the effects of wearing a denture on chewing efficacy could be determined from the viewpoint of cognitive functioning and related oral structural environment in partially edentulous elderly subjects by evaluating prefrontal and chewing activities while chewing with and without wearing a denture, and then comparing the results with those of young healthy subjects. The present study was conducted to investigate masticatory muscle and prefrontal activities during chewing, as well as occlusal force and masticatory score under the conditions of wearing a denture and tooth loss.

Masticatory muscle activities are generated by cortical and brainstem sensorimotor systems including peripheral couplings [36–38], while masticatory score is a subjective evaluation of masticatory ability [39, 40]. Furthermore, the prefrontal activity participates in higher cognition for chewing performance [28–32, 41]. Therefore, we used a systematic approach to elucidate the effects of wearing a denture on chewing-related prefrontal cognition and chewing activity by the occlusal force and masticatory muscles, and also performed a subjective evaluation of chewing ability using masticatory score in partially edentulous elderly patients.

Functional near-infrared spectroscopy (fNIRS) has recently been used to examine cognitive state in aged individuals [42–44], as well as in those with dementia [45], Alzheimer’s disease [46–48], and psychiatric disorders [49–54]. Hence, we considered that prefrontal chewing cognition in elderly edentulous subjects could be evaluated by this technique during chewing performance with and without use of a denture prosthesis. fNIRS is considered to be suitable for examinations of prefrontal activities during chewing performance with and without wearing a denture in clinical conditions, as several previous studies utilized it to detect prefrontal activities during jaw clenching [32] and chewing [28, 55, 56]. In the present study, we attempted to determine the qualities of prefrontal activities in partially edentulous elderly individuals during chewing while wearing a denture as compared with young healthy controls. This is the first known study to explore prefrontal function during chewing in partially edentulous elderly subjects to determine the efficacy of wearing a denture.

## Materials and Methods

### Subjects

**Fig 1:**
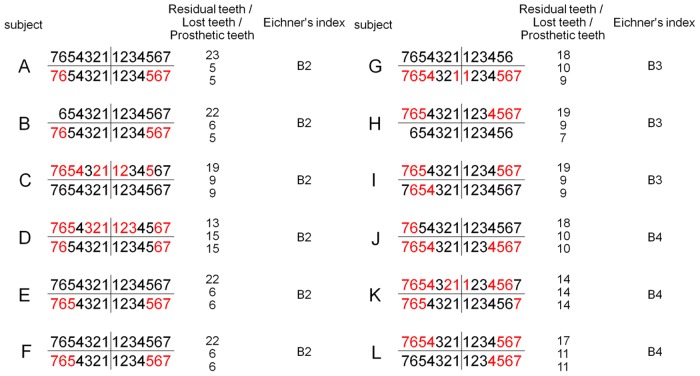
Dental state of 12 partially edentulous subjects. Shown is the dental state of each of the present 12 partially edentulous subjects (A to L). Using Eichner’s tooth loss classification, subjects A-F were classified as B2, G-I were classified as B3, and J-L were classified as B4. The mean numbers of residual, lost, and prosthetic teeth in those groups were 18.8±3.16, 9.2±3.16, and 8.8±3.3, respectively. Residual teeth are indicated in black and prosthetic teeth in red.

Twelve partially edentulous subjects [6 males, 6 females; 63.1±6.1 (mean ± standard deviation (SD) years old] with removable partial dentures and being followed at the Prosthodontics Department of Nihon University School of Dentistry at Matsudo Hospital, and 12 healthy young volunteers (6 males, 6 females; 22.1±2.3 years old) recruited from staff members of the Nihon University School of Dentistry at Matsudo were enrolled. The sample size was determined using the G*Power 3 software package (noncommercial program downloaded from University of Dusseldorf, Germany) [57], which established parameters with a significance level of 0.05, statistical power of 0.8, and effect size of 0.25 (medium effect). Since the necessary minimum sample size was shown to be 5, we decided to recruit at least 12 participants in order to detect significant differences. As for the partially edentulous subjects, they were divided into 3 groups based on Eichner’s tooth loss classification (B2, n = 6; B3, n = 3; B4, n = 3) [58]. Eichner’s index is based on the existence of occlusal contact between premolars and molars, which is called the supporting zone, with 2 supporting zones in B2, 1 in B3, and anterior tooth contact with no supporting zones in B4. The mean numbers of residual, lost, and prosthetic teeth in the present partially edentulous subjects were 18.8±3.16, 9.2±3.16, and 8.8±3.3, respectively (Fig 1 and Table 1). All subjects in the young group had complete natural dentition, while all subjects in both groups were right handed and mentally healthy. None had any symptoms of temporomandibular joint or masticatory muscle dysfunctions, such as pain and difficulties, and none of the partially edentulous subjects had complaints regarding their dentures. Each subject provided written informed consent for participation in the study, which was approved by the Ethics Committee of Nihon University School of Dentistry at Matsudo (EC06-008).

**Table 1:** Numbers of residual, lost, and prosthetic teeth in 12 elderly edentulous subjects.

Residual teeth [mean (SD)]	Lost teeth [mean (SD)]	Prosthetic teeth [mean (SD)]
18.8 (3.16)	9.2 (3.16)	8.8 (3.3)

### Experimental procedures

During the 3 months prior to the study task, the partially edentulous subjects received sufficient prosthodontics treatments in the form of conventional removable partial denture prostheses, including artificial teeth, a denture base, clasp retainers, major and minor connectors, and rest under a cross-arch stabilized condition. None had complaints about the prosthesis, such as discomfort, pain, or difficulties in chewing performance. A 35-item food intake questionnaire method was used to evaluate their chewing ability [37, 38]. Masticatory muscle and prefrontal activities were recorded in a quiet room with the subject comfortably seated so that their Frankfurt horizontal plane was parallel to the floor. Using fNIRS and electromyography, we simultaneously recorded those activities in the partially edentulous subjects while wearing a removable partial denture (Wearing Denture) and without wearing a denture (Tooth Loss), as well as in the young subjects (Young) while performing a unilateral gum chewing task.

### Masticatory score

As a self-assessment of chewing ability, food intake was queried according to a method previously described by Hirai et al. [37, 38]. Based on answers to a questionnaire, mastication scores were determined in order to evaluate chewing activity. The subjects rated their ability to chew 35 different food items according to the following scale: 2, can be eaten easily; 1, can be eaten with difficulty; 0, cannot be eaten, with the 35 foods classified into 5 grades of difficulty based on the scores, as follows: Group I, pudding, bananas, boiled cabbage, boiled carrots, boiled taro, sliced raw tuna, boiled onions; Group II, strawberries, ham, boiled chicken, boiled fish paste patty, *konnyaku*, boiled kelp (*tsukudani kombu*), raw cucumber; Group III, fried chicken, fried rice crackers, roasted chicken, apples, pickled eggplants, boiled beef, raw cabbage; Group IV, roasted pork, pickled scallion, pickled radish, rice cake, peanuts, sliced raw cuttlefish, pork cutlet; and Group V, raw carrots, pickled radish, jellyfish, vinegar octopus, raw sea cucumber, raw abalone, dried cuttlefish. Masticatory score was calculated as follows: (total of Group I + total of Group II × 1.14 + total of Group III × 1.30 + total of Group IV × 1.52 + total of Group V × 3.00) × 100/111.4. In the present study, a mastication score greater than 80% was considered to be normal [37].

### Task

We examined prefrontal activities in the subjects using fNIRS during 5 trials performed during the chewing session, as well as during the rest (Rest session). A typical chewing session consisted of 5 chewing trials, each conducted for 10 sec with unilateral gum chewing. Each of the 5 trials was separated from the succeeding trial by a 40-sec rest phase. For the chewing task, we used 1 piece of chewing gum (Freezone, Lotte Co., Japan), and the start and end of each trial was indicated to the participant by verbal commands. The subjects were instructed to be quiet until given a verbal cue (pre-chewing period), then after given a verbal cue to start, they began to chew the gum for 10 sec (chewing period) until a verbal cue to stop chewing. Both the partially edentulous and Young were instructed to chew equally on the right and left sides during a chewing session, and to avoid head movements during performance of the task. Further, the chewing sessions were conducted under 2 different conditions, Tooth Loss and Wearing Denture, in the partially edentulous subjects. In all subjects, fNIRS measurements were also performed during the Rest session, during which there was no task performance, and the partially edentulous subjects performed Rest sessions under both the Tooth Loss and Wearing Denture conditions. To avoid the influence of task sequence on the results, the subjects were asked to perform the tasks in a randomized sequence.

### Maximal occlusal force

Bilateral maximal occlusal force was measured with 97-μm thick pressure sensitive sheets (Dental Prescale 50H Rtype, Fuji Film Co., Tokyo, Japan). The subjects performed maximal clenching in the intercuspal position with a sheet placed between the maxillary and mandibular dental arch. Subjects with removable partial dentures kept their appliances in place during measurement of maximal occlusal force. Bite force was calculated by color development using pressure-sensitive film with special analytical equipment (Occluzer FPD703, Fuji Film Co., Tokyo, Japan).

### Measurement of masticatory muscle EMG activity during chewing performance

Masticatory muscle activity was recorded while chewing using surface EMG electrodes. After cleaning the skin with ethanol, a pair of bipolar Ag/AgCl electrodes, each 7 mm in diameter, was attached to skin overlying the corresponding muscle. The electrodes were positioned bilaterally on the center of the masseter (Mm, jaw closing), anterior temporal (Ta, jaw closing), and anterior digastric (AD, jaw opening) muscles parallel with the direction of the muscle fibers, with an inter-electrode distance of 20 mm. A ground electrode was attached to the left ear lobe. EMG signals were amplified (POLYGRAPH BIOELECTRIC AMPL 1253A, San-ei MED, Tokyo, Japan), with the high frequency cut-off filter set at 1 kHz and a time constant of 0.03 sec. Amplified EMG signals were digitized with 16-bit resolution by use of an A/D converter (APA16-32/2(OB) F, CONTEC, Tokyo, Japan) and then downloaded to a personal computer at a sampling rate of 1 kHz. We analyzed the cycle duration of AD muscle EMG activities, burst duration, mean integrated area (Area), and peak amplitude of the EMG activities of the Mm, Ta, and AD muscles during each chewing cycle.

### fNIRS measurement during chewing performance

Prefrontal activity was assessed during the pre-chewing, chewing, and post-chewing periods using a 22-channel fNIRS device (ETG-100, Hitachi Medical Co., Chiba, Japan), which utilizes near-infrared light at 2 wavelengths, 780 and 830 nm [26]. The distance between each pair of detector probes was 3.0 cm and the device was set to measure at points associated with the surface of the cerebral cortex [59, 60]. The probes were fitted with 3×5 thermoplastic shells and placed in the prefrontal region, while the bottom lines of the fNIRS probes were set according to FP1 and FP2, with referral to the international 10–20 system [61]. fNIRS was used to determine relative changes in concentrations of oxygenated-hemoglobin ([oxy-Hb]), deoxygenated-hemoglobin ([deoxy-Hb]), and total hemoglobin ([total-Hb]), with fundamental Hb data displayed and used as an index of Hb change as a scaled variable. Change in [oxy-Hb] was used as an indicator of change in regional cerebral blood volume, as that has been reported to be more sensitive than [deoxy-Hb] as a parameter for measuring blood flow change associated with brain activation [62] and has a strong correlation with blood-oxygenation-level-dependent signals measured by fMRI [63]. The sampling interval was 0.1 sec. During the measurements, the subjects were instructed to open their eyes and gaze at a point in front of them. Each trial was repeated 5 times and obtained values were averaged using the ‘integral mode’ of the ETG-100 software for both the chewing (Tooth Loss and Wearing Denture conditions in partially edentulous subjects) and Rest sessions. The moving average method (moving average window, 5 sec) was used to exclude short-term motion artifacts. Measurement baselines were corrected using linear fitting [64], which was performed by connecting the pre-chewing baseline (mean of final 20 sec of pre-chewing period) to the post-chewing baseline (mean of final 20 sec of post-chewing period). Channels 14 and 18 presented a decrease in [oxy-Hb] preceding chewing task performance, indicating the appearance of [oxy-Hb] artifacts caused by preliminary strain related to temporal muscle and jaw movement activities. Thus, those channels were excluded from the fNIRS measurements.

### Anatomical localization of fNIRS channels

**Fig 2:**
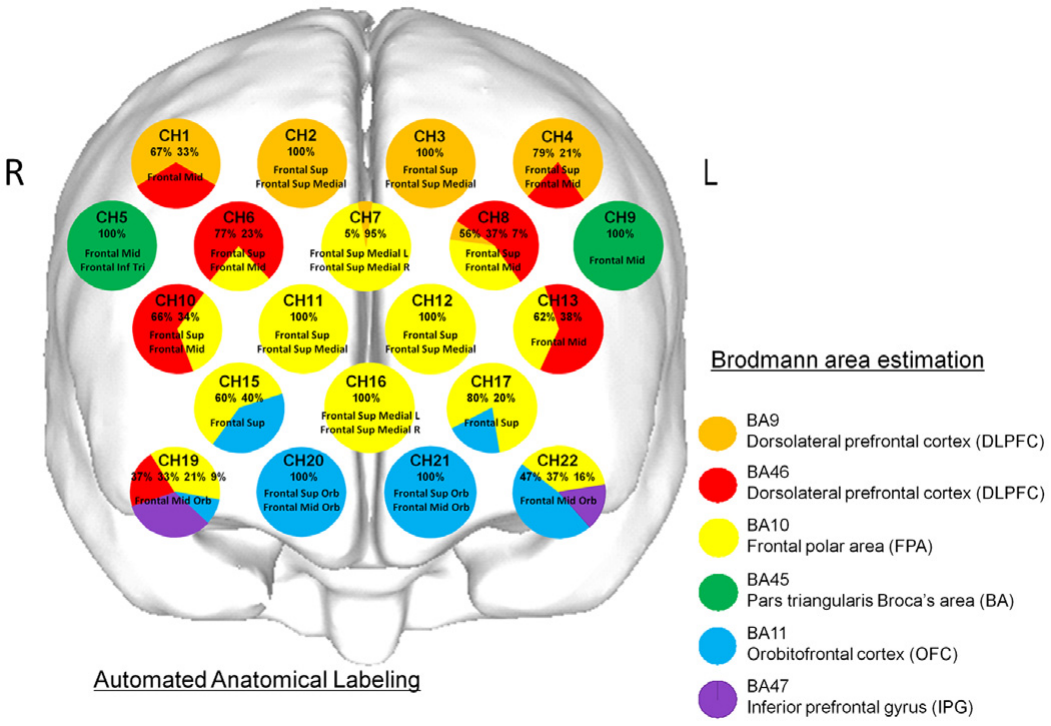
Anatomical identification of near-infrared spectroscopy channels. The coordinates for all probe and anatomical landmark positions (Nz, Iz, A1, A2, Cz) were obtained using a 3-dimensional digitizer. Probabilistic registration was used to transcribe the measuring points for each subject according to the protocol of the Montreal Neurological Institute and those points were projected onto the cortical surface. Anatomical localization was identified using the Platform for Optical Topography Analysis Tools, with reference to the Automated Anatomical Labeling system. Orange, red, yellow, green, blue, and purple represent DLPFC (BA9), DLPFC (BA46), FPA (BA10), BA (BA45), OFC (BA11), and IPG (BA47), respectively. Each circle corresponds to a channel and the pie chart within each circle shows the percentages of areas in that channel.

The coordinates for all probe and anatomical landmark (Nz, Iz, A1, A2, Cz) positions were obtained using a three-dimensional digitizer (3SPACE ISOTRAK2, Polhemus, US) and transcribed into Montreal Neurological Institute standard brain space [65, 66] using probabilistic registration [67, 68]. Probe positions were then projected onto the cortical surface and the anatomic localization corresponding to each probe coordinate was identified using Platform for Optical Topography Analysis Tools (POTATo, Hitachi, Japan), with reference to the Brodmann area [67–69] (Fig 2).

### Statistical analysis

For comparing masticatory scores, occlusal force, and masticatory muscle activities (cycle duration, burst duration, area, peak amplitude) between the Tooth Loss and Wearing Denture conditions in the partially edentulous subjects, we used a paired t-test if normality was passed, while a Wilcoxon Signed Rank test was used if normality failed. We also utilized one-way analysis of variance (ANOVA) and Dunnett’s method for those comparisons when the normality test was passed, and Kruskal-Wallis one-way ANOVA on ranks and Dunn’s method if normality failed.

Values for [oxy-Hb] were calculated every 1 sec and compared between the Tooth Loss condition and Rest session, and the Wearing Denture condition and Rest session in the partially edentulous subjects, and between the Young condition and Rest session in the controls. For a comparison of values for [oxy-Hb] between Tooth Loss and Wearing Denture while chewing, we used a paired t-test, while we used Student’s t-test to compare between Tooth Loss and Young, and between Wearing Denture and Young while chewing. All statistical analyses were implemented as part of a plug-in-based analysis platform that runs on MATLAB (The MathWorks Inc., MA, USA). A topographical representation of significant (p<0.01) channels monitored every 1 sec was projected onto the frontal lobe surface of Montreal Neurological Institute standard brain space [70, 71] using a 3-dimensional composite display unit (version 2.41, Hitachi Medical Co., Chiba, Japan) [72].

It has been reported that as the number of items being examined increases, so does the risk of type 1 errors [73]. Thus, in order to avoid such errors, two-way repeated measures ANOVA and multiple comparisons using a Bonferroni t-test were applied to the time course of data for [oxy-Hb] obtained every 1 sec during the pre-chewing, chewing, and post-chewing periods under the Tooth Loss and Wearing Denture conditions, then two-way measures ANOVA and multiple comparisons versus the control group (Young) were applied using a Bonferroni t-test to compare between Tooth Loss and Young, and between Wearing Denture and Young. The statistical software package SigmaPlot 12.5 (Systat Software Inc., CA, USA) was used for all analyses and p values less than 0.05 were considered to be statistically significant. Results are expressed as the mean ± SD, p-value, or power of the performed test.

## Results

### 1. Masticatory score

The masticatory score for the Tooth Loss condition (48.0±22.1) was significantly (paired t-test, p<0.001, power of performed test = 0.998) lower than that for the Wearing Denture condition (81.1±11.7). Furthermore, masticatory scores for both Tooth Loss and Wearing Denture were significantly (Kruskal-Wallis one-way ANOVA on ranks, p<0.001; Dunn’s method, p<0.05) lower than for Young (98.5±2.4) (Table 2).

**Table 2:** Masticatory scores for Tooth Loss, Wearing Denture, and Young.

Tooth Loss [mean (SD)]	Wearing Denture [mean (SD)]	Young [mean (SD)]
48.0 (22.1) **a	81.1 (11.7) b	98.5 (2.4)

The score for Tooth Loss was significantly decreased as compared with Wearing Denture (*p*<0.01, **paired t-test). Significant differences were found among Tooth Loss, Wearing Denture, and Young [*p*<0.05, Kruskal-Wallis one-way ANOVA on ranks and multiple comparisons versus control group (Young), Dunn's method, a: Tooth Loss vs. Young, b: Wearing Denture vs. Young].

### 2. Occlusal force

Occlusal force for Tooth Loss (304.8±104.2 N) was significantly (paired t-test, p<0.001, power of performed test = 1.0) lower than that for Wearing Denture (638.7±127.0 N) and also significantly (Kruskal-Wallis one-way ANOVA on ranks, p<0.001 and Dunn’s method, p<0.05) lower than that for Young (1200.7±642.3 N) (Table 3).

**Table 3:** Occlusal force (N) for Tooth Loss, Wearing Denture, and Young.

Tooth Loss [mean (SD)]	Wearing Denture [mean (SD)]	Young [mean (SD)]
304.8 (104.2) **a	638.7 (127.0)	1200.7 (642.3)

Occlusal force for Tooth Loss was significantly decreased as compared with Wearing Denture (*p*<0.01, **paired t-test). A significant difference was found between Tooth Loss and Young [*p*< 0.05, Kruskal-Wallis one-way ANOVA on ranks and multiple comparisons versus control group (Young), Dunn's method, a: Tooth Loss vs. Young].

### 3. Masticatory muscle EMG activities

The cycle durations for AD EMG activities for Tooth Loss (857.0±153.2 msec) and Wearing Denture (834.8±161.3 msec) were significantly (Kruskal-Wallis one-way ANOVA on ranks, p<0.001; Dunn’s method, p<0.05) longer as compared to the Young (669.2±71.5 msec) (Table 4).

**Table 4:** Masticatory muscle EMG activities for Tooth Loss, Wearing Denture, and Young.

Electromyography	Tooth Loss [mean (SD)]	Wearing Denture [mean (SD)]	Young [mean (SD)]
Cycle duration (msec)			
AD	857.0 (153.2) a	834.8 (161.3) b	669.2 (71.5)
Burst duration (msec)			
Mm	212.1 (60.3) **	327.9 (75.2)	268.4 (24.7)
Ta	222.1 (83.8) **	305.4 (67.8)	245.6 (46.5)
AD	338.8 (69.9)	313.4 (41.3)	300.1 (53.0)
Area (mV·sec)			
Mm	0.011 (0.006) **††** c	0.026 (0.017)	0.036 (0.018)
Ta	0.009 (0.004) **††** c	0.017 (0.010)	0.025 (0.012)
AD	0.010 (0.004)	0.013 (0.003)	0.013 (0.007)
Peak amplitude (mV)			
Mm	0.12 (0.13) b	0.15 (0.09)	0.28 (0.15)
Ta	0.09 (0.09) b	0.15 (0.09)	0.19 (0.09)
AD	0.19 (0.07)	0.23 (0.07)	0.24 (0.19)

Masticatory muscle EMG activities for Tooth Loss were significantly decreased as compared with Wearing Denture (*p*<0.01, **paired t-test, ††Wilcoxon signed Rank Test). Furthermore, significant differences were found among Tooth Loss, Wearing Denture, and Young [*p*<0.01, Kruskal-Wallis one-way ANOVA on ranks and multiple comparisons versus control group (Young), Dunn's method, a: Tooth Loss vs. Young, b: Wearing Denture vs. Young; *p*<0.05, one-way ANOVA and multiple comparisons versus control group (Young), Dunnett’s method, c: Tooth Loss vs. Young].

The burst duration for Mm EMG activity for Tooth Loss (212.1±60.3 msec) was significantly (paired t-test, p<0.001, power of performed test = 1.0) shorter than that for Wearing Denture (327.9±60.3 msec) (Table 4). Furthermore, the burst duration for Ta EMG activity for Tooth Loss (305.4±67.8 msec) was significantly (paired t-test, p<0.01, power of performed test = 0.784) shorter as compared to Wearing Denture (244.1±57.6 msec) (Table 4).

The areas for Mm (0.011±0.006 mV·sec) and Ta (0.009±0.004 mV·sec) EMG activities for Tooth Loss were significantly (Wilcoxon Signed Rank test, p<0.001) lower than those for Wearing Denture (Mm; 0.026±0.017 mV·sec, Ta; 0.017±0.010 mV·sec) (Table 3) and also significantly (one-way ANOVA, p<0.001, Dunnett’s method, p<0.05) lower as compared to Young (Mm; 0.036±0.018 mV·sec, Ta; 0.025±0.012 mV·sec) (Table 4).

The peak amplitude values for Mm (0.12±0.13 mV) and Ta (0.09±0.09 mV) EMG activities for Tooth Loss were significantly (Kruskal-Wallis one-way ANOVA on ranks, p<0.01; Dunn’s method, p<0.05) lower than those for Mm (0.28±0.15 mV) and Ta (0.19±0.09 mV) for Young (Table 4).

### 5. Cross-sectional statistical topography of [oxy-Hb] for Tooth Loss and Rest

**Fig 3:**
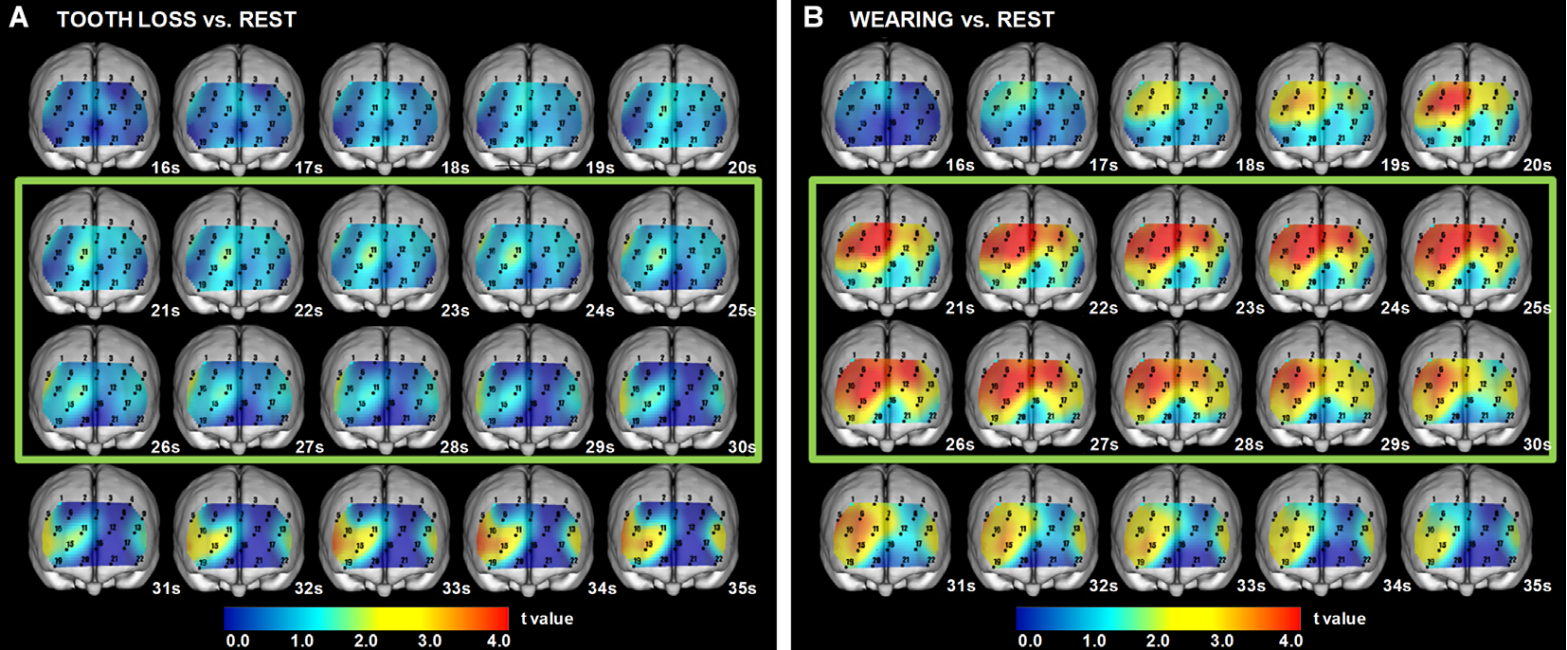
Cross-sectional statistical topography under Wearing Denture and Tooth Loss conditions, as compared to Rest session. (A) The values for [oxy-Hb] under the Tooth Loss were significantly increased (paired t-test, p<0.05), as compared to those in the Rest session, during the chewing period for BA (BA45), and the post-chewing period for DLPFC (BA46), FPA (BA10), BA (BA45), and OFC (BA11). Green square indicates chewing period. Paired t-test results showed a 5% risk rate value of 2.20 and 1% risk rate value of 3.11. (B) The values for [oxy-Hb] under the Wearing Denture were significantly increased (paired t-test, p<0.05), as compared to those in the Rest session, during the pre-chewing period for DLPFC (BA9, BA46) and FPA (BA10), during the chewing period for DLPFC (BA9, BA46), FPA (BA10), BA (BA45), OFC (BA11), and IPG (BA47), and during the post-chewing period for DLPFC (BA9, BA46), FPA (BA10), BA (BA45), OFC (BA11), and IPG (BA47). Green square indicates chewing period. Paired t-test results showed a 5% risk rate value of 2.20 and 1% risk rate value of 3.11.

As compared to the Rest session, the values for [oxy-Hb] under the Tooth Loss condition were not significantly (paired t-test, p<0.05) different during the pre-chewing period. In contrast, as compared to Rest session, the values for [oxy-Hb] under Tooth Loss were significantly (paired t-test, p<0.05) increased during the chewing period for BA (BA45, ch. 5), and during the post-chewing period for DLPFC (BA46, ch. 10, 13), FPA (BA10, ch. 10, 11, 13, 15), BA (BA45, ch. 5), and OFC (BA11, ch. 15) (Fig 3A and Table 5-1).

**Table 5:** Significantly different channels and corresponding brain regions presented in cross-sectional statistical topography of [oxy-Hb].

**1. Tooth Loss vs. Rest**			
	Pre-chewing	Chewing	Post-chewing
DLPFC	ns	ns	ch. 10, 13
FPA	ns	ns	ch. 10, 11, 13, 15
BA	ns	ch. 5	ch. 5
OFC	ns	ns	ch. 15
IPG	ns	ns	ns
**2. Wearing Denture vs. Rest**			
	Pre-chewing	Chewing	Post-chewing
DLPFC	ch. 1, 2, 4, 6, 7, 8, 10	ch. 1, 2, 3, 4, 6, 7, 8, 10, 13,19	ch. 1, 2, 6, 7, 10, 13, 19
FPA	ch. 6, 7, 8, 10, 11	ch. 6, 7, 8, 10, 11, 12, 13, 15, 17, 19	ch. 6, 7, 10, 11, 15, 19
BA	ns	ch. 5, 9	ch. 5, 9
OFC	ns	ch. 15, 17, 19	ch. 15, 19
IPG	ns	ch. 19	ch. 19
**3. Young vs. Rest**			
	Pre-chewing	Chewing	Post-chewing
DLPFC	ch. 6, 10, 13, 19	ch. 1, 2, 3, 4, 6, 7, 8, 10, 13, 19	ch. 1, 2, 6, 10, 13, 19
FPA	ch. 6, 10, 11, 12, 13, 15, 16, 17, 19	ch. 6, 7, 8, 10, 11, 12, 13, 15, 16, 17, 19, 22	ch. 6, 10, 11, 13, 16, 19, 22
BA	ch. 5	ch. 5, 9	ch. 5, 9
OFC	ch.15, 17, 20, 21	ch. 15, 17, 19, 20, 21, 22	ch. 19, 22
IPG	ns	ch. 19, 22	ch. 19, 22

The channels shown presented significantly (paired t-test, p<0.05) increased values for [oxy-Hb] between Tooth Loss and Rest, Wearing Denture and Rest, and Young and Rest. Chewing under Tooth Loss activated only 1 channel in the prefrontal cortex, while Wearing Denture and Young showed numerous markedly activated channels in the prefrontal cortex as compared with the Rest condition.

### 6. Cross-sectional statistical topography of [oxy-Hb] for Wearing Denture and Rest

As compared to the Rest session, the values for [oxy-Hb] under the Wearing Denture condition were significantly (paired t-test, P<0.05) increased during the pre-chewing period for DLPFC (BA9, BA46, ch. 1, 2, 4, 6, 7, 8, 10), and FPA (BA10, ch. 6, 7, 8, 10, 11), and during the chewing period for DLPFC (BA9, BA46, ch. 1, 2, 3, 4, 6, 7, 8, 10, 13,19), FPA (BA10, ch. 6, 7, 8, 10, 11, 12, 13, 15, 17, 19), BA (BA45, ch. 5, 9), OFC (BA11, ch. 15, 17, 19), and IPG (BA47, ch. 19), and during the post-chewing period for DLPFC (BA9, BA46, ch. 1, 2, 6, 7, 10, 13, 19), BA (BA45, ch. 5, 9), FPA (BA10, ch. 6, 7, 10, 11, 15, 19), OFC (BA11, ch. 15, 19), and IPG (BA47, ch. 19) (Fig 3B and Table 5-2).

### 7. Temporal changes in [oxy-Hb] for Tooth Loss and Wearing Denture

**Fig 4:**
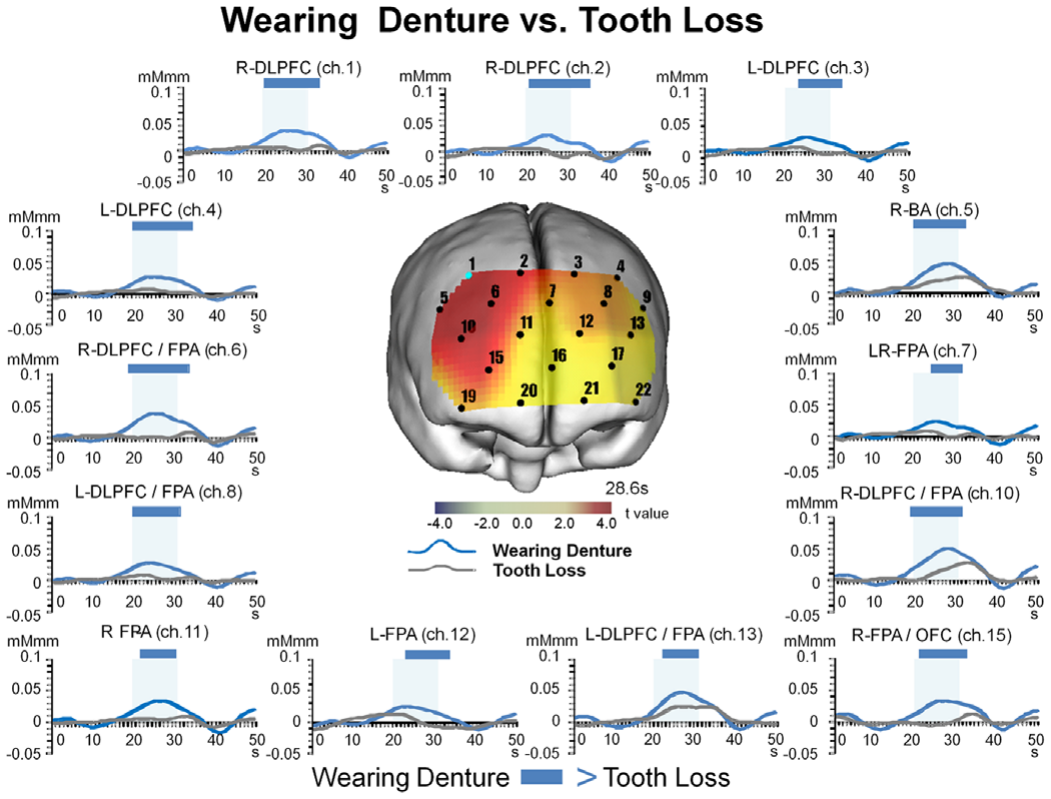
Temporal changes under Tooth Loss and Wearing Denture conditions. The values for [oxy-Hb] during the pre-chewing period for Wearing Denture were significantly (two-way repeated measures ANOVA and Bonferroni t-test, p<0.05) increased for DLPFC (BA46), and FPA (BA10), and during the chewing period for DLPFC (BA9, BA46), FPA (BA10), BA (BA45), and OFC (BA11), and remained increased during the post-chewing period for DLPFC (BA9, BA46), FPA (BA10), BA (BA45), and OFC (BA11) as compared to Tooth Loss. Significant differences between the conditions are indicated by a blue bar.

Based on the results of statistical comparisons between values obtained under the Tooth Loss and Wearing Denture conditions (paired t-test), we performed two-way repeated measures ANOVA using data for [oxy-Hb] obtained from all channels, with the results shown in Fig 4.

Our results showed significant interactions of Wearing Denture and Tooth Loss with time in ch. 1 (F = 4.136, p<0.001, power of performed test = 1.0), ch. 2 (F = 3.921, p<0.001, power of performed test = 1.0), ch. 3 (F = 2.747, p<0.001, power of performed test = 0.999), ch. 4 (F = 3.855, p<0.001, power of performed test = 1.0), ch. 5 (F = 3.372, p<0.001, power of performed test = 1.0), ch. 6 (F = 5.219, p<0.001, power of performed test = 1.0), ch. 7 (F = 1.773, p<0.001, power of performed test = 0.8939), ch. 8 (F = 2.440, p<0.001, power of performed test = 0.988), ch. 10 (F = 3.976, p<0.001, power of performed test = 1.0), ch. 11 (F = 1.977, p<0.001, power of performed test = 0.9668), ch. 12 (F = 2.153, p<0.001, power of performed test = 0.9896), ch. 13 (F = 2.312, p<0.001, power of performed test = 0.9968), and ch. 15 (F = 2.957, p<0.001, power of performed test = 1.0).

When Wearing Denture was compared to Tooth Loss, we found significant (Bonferroni t-test, p<0.05) increases in the values for [oxy-Hb] during the pre-chewing period for DLPFC (BA46, ch. 6, 10) and FPA (BA10, ch. 6, 10), during the chewing period for DLPFC (BA9, BA46, ch. 1, 2, 3, 4, 6, 8, 10, 13), FPA (BA10, ch. 6, 7, 8, 10, 11, 12, 13, 15), OFC (BA11, ch. 15), and BA (BA45, ch. 5), and during the post-chewing period for DLPFC (BA9, BA46, ch. 1, 2, 3, 4, 6, 8, 10), FPA (BA10, ch. 6, 7, 8, 10, 12, 15), OFC (BA11, ch. 15), and BA (BA45, ch. 5) (Fig 4 and Table 6-1).

**Table 6:** Significantly different channels and corresponding brain regions presented in temporal changes of [oxy-Hb].

**1. Tooth Loss vs. Wearing Denture**			
	Pre-chewing	Chewing	Post-chewing
DLPFC	ch. 6, 10	ch. 1, 2, 3, 4, 6, 8, 10, 13	ch. 1, 2, 3, 4, 6, 8, 10
FPA	ch. 6, 10	ch. 6, 7, 8, 10, 11, 12, 13, 15	ch. 6, 7, 8, 10, 12, 15
BA	ns	ch. 5	ch. 5
OFC	ns	ch. 15	ch. 15
IPG	ns	ns	ns
**2. Tooth Loss vs. Young**			
	Pre-chewing	Chewing	Post-chewing
DLPFC	ns	ch. 1, 2, 4, 6, 10	ch. 1, 2, 6, 10
FPA	ch. 15	ch. 6, 10, 11, 12, 15	ch. 6, 10
BA	ns	ch. 5	ch. 5
OFC	ch. 15	ch. 15	ns
IPG	ns	ns	ns
**3. Wearing Denture vs. Young**			
	Pre-chewing	Chewing	Post-chewing
DLPFC	ns	ch. 1, 6	ch. 6
FPA	ns	ch. 6	ch. 6
BA	ns	ch. 5	ns
OFC	ns	ns	ns
IPG	ns	ns	ns

The channels shown presented significantly (Bonferroni t-test, p<0.05) changed values for [oxy-Hb] between Tooth Loss and Wearing Denture, Tooth Loss and Young, and Wearing Denture and Young. During the chewing period under both the Young and Wearing Denture conditions, there was a large number of activated channels in the prefrontal cortex as compared with Tooth Loss, while there were few activated channels in the prefrontal cortex during that period under the Wearing condition as compared with Young.

### 8. Cross-sectional statistical topography of [oxy-Hb] for Young and Rest

**Fig 5:**
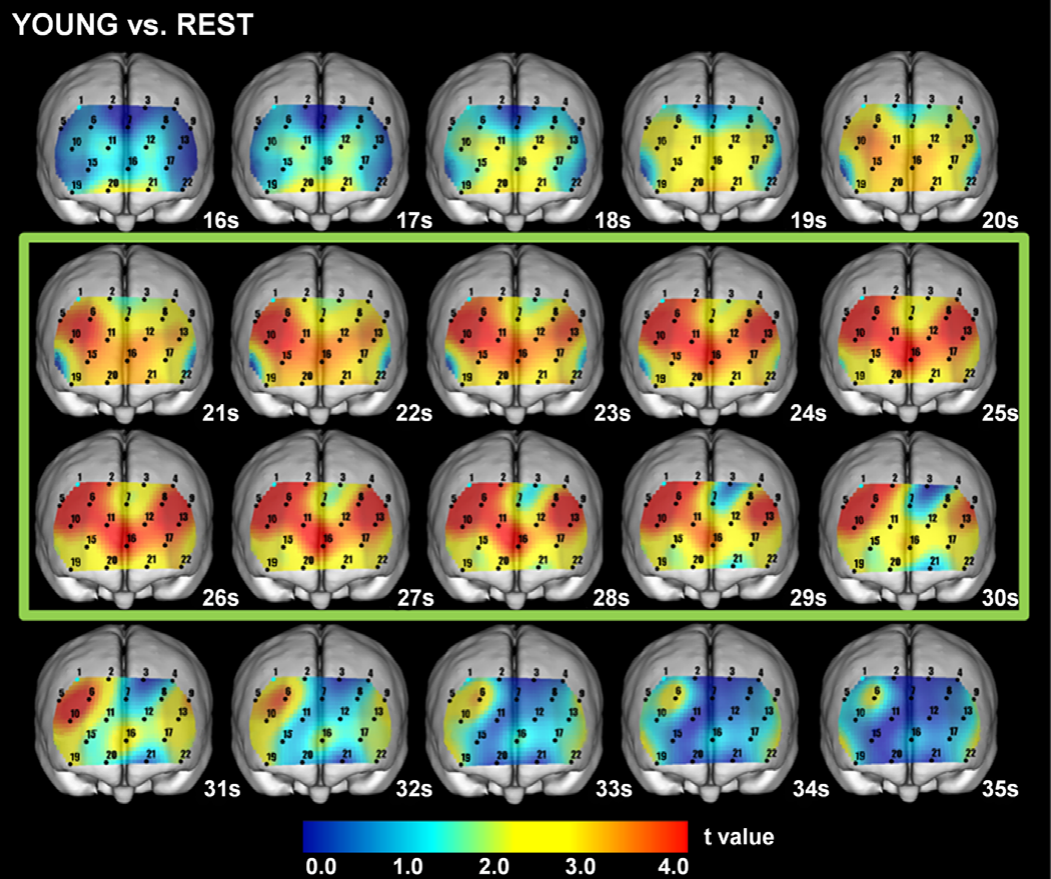
Cross-sectional statistical topography in Young as compared to Rest session. The values for [oxy-Hb] in the Young were significantly (paired t-test, p<0.05) increased, as compared to those in the Rest session, during the pre-chewing period for DLPFC (BA9, BA46), FPA (BA10), BA (BA45), and OFC (BA11), during the chewing period for DLPFC (BA9, BA46), FPA (BA10), BA (BA45), OFC (BA11), and IPG (BA47), and during the post-chewing period for DLPFC (BA9, BA46), FPA (BA10), BA (BA45), OFC (BA11), and IPG (BA47). Green square indicates chewing period. Paired t-test results showed a 5% risk rate value of 2.20 and 1% risk rate value of 3.11.

As compared to the Rest session, the values in the Young for [oxy-Hb] were significantly (paired t-test, p<0.05) increased during the pre-chewing period for DLPFC (BA46, ch. 6, 10, 13, 19), FPA (BA10, ch. 6, 10, 11, 12, 13, 15, 16, 17, 19), BA (BA45, ch. 5), and OFC (BA11, ch.15, 17, 20, 21), during the chewing period for DLPFC (BA9, BA46, ch. 1, 2, 3, 4, 6, 7, 8, 10, 13, 19), FPA (BA10, ch. 6, 7, 8, 10, 11, 12, 13, 15, 16, 17, 19, 22), BA (BA45, ch. 5, 9), OFC (BA11, ch. 15, 17, 19, 20, 21, 22), and IPG (BA47, ch. 19, 22), and during the post-chewing period for DLPFC (BA9, BA46, ch. 1, 2, 6, 10, 13, 19), FPA (BA10, ch. 6, 10, 11, 13, 16, 19, 22), BA (BA45, ch. 5, 9), OFC (BA11, ch. 19, 22), and IPG (BA47, ch. 19, 22) (Fig 5 and Table 5-3).

### 9. Temporal changes in [oxy-Hb] for Tooth Loss and Wearing Denture, as compared to Young

Based on our findings obtained using two-way repeated measures ANOVA of comparisons between the Tooth Loss and Wearing Denture conditions, we also performed two-way repeated measures ANOVA of [oxy-Hb] data for Tooth Loss and Wearing Denture, as compared those with Young.

There were significant interactions found between both conditions (Tooth Loss, Wearing Denture) in the partially edentulous subjects and the Young in regard to time in ch. 1 (F = 2.475, p<0.001, power of performed test = 1.0), ch. 2 (F = 2.442, p<0.001, power of performed test = 1.0), ch. 4 (F = 1.256, p<0.05, power of performed test = 0.4906), ch. 5 (F = 1.791, p<0.001, power of performed test = 0.9944), ch. 6 (F = 4.113, p<0.001, power of performed test = 1.0), ch. 10 (F = 1.461, p<0.05, power of performed test = 0.8513), ch. 11 (F = 2.107, p<0.001, power of performed test = 0.999), ch. 12 (F = 2.071, p<0.001, power of performed test = 0.999), and ch. 15 (F = 2.569, p<0.001, power of performed test = 1.0). In contrast, there were no significant interactions found between either of those conditions in the partially edentulous subjects and the Young in ch. 3, 7, 8, and 13.

#### 9.1. Temporal changes in [oxy-Hb] for Tooth Loss as compared to Young

**Fig 6:**
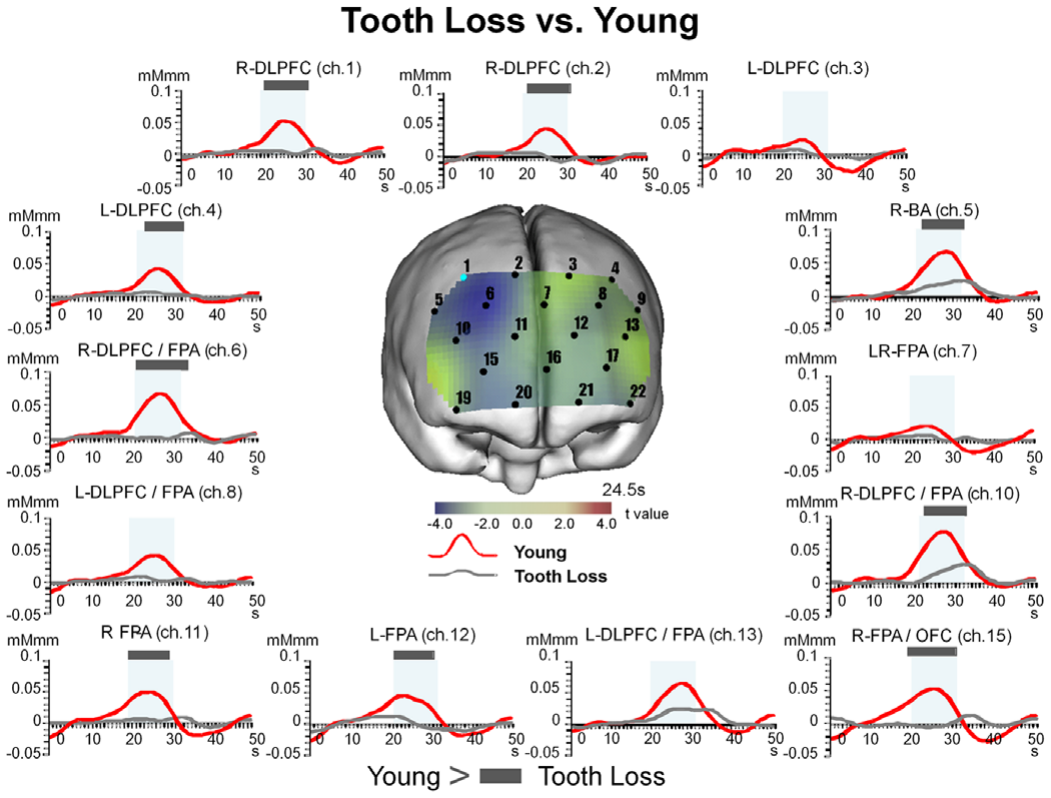
Temporal changes in [oxy-Hb] under Tooth Loss condition as compared to Young. The values for [oxy-Hb] and under the Tooth Loss were significantly (two-way repeated measures ANOVA and Bonferroni t-test, p<0.05) decreased as compared to those in the Young group during the pre-chewing period for FPA (BA10) and OFC (BA11), during the chewing period for DLPFC (BA9, BA46), FPA (BA10), BA (BA45), and OFC (BA11), and during the post-chewing period for DLPFC (BA9, BA46), FPA (BA10), and BA (BA45). Significant differences between Tooth Loss and Young are indicated by a grey bar. All channels shown here correspond to channels that showed significant differences between the Wearing Denture and Tooth Loss presented in Fig 4.

When we compared the Tooth Loss with the Young, we found significant (Bonferroni t-test, p<0.05) decreases in the values for [oxy-Hb] during the pre-chewing period for FPA (BA10, ch. 15) and OFC (BA11, ch. 15), during the chewing period for DLPFC (BA9, BA46, ch. 1, 2, 4, 6, 10), FPA (BA10, ch. 6, 10, 11, 12, 15), BA (BA45, ch. 5), and OFC (BA11, ch. 15), and during the post-chewing period for DLPFC (BA9, BA46, ch. 1, 2, 6, 10), FPA (BA10, ch. 6, 10), and BA (BA45, ch. 5) (Fig 6 and Table 6-2).

#### 9.2. Temporal changes in [oxy-Hb] for Wearing Denture as compared to Young

**Fig 7:**
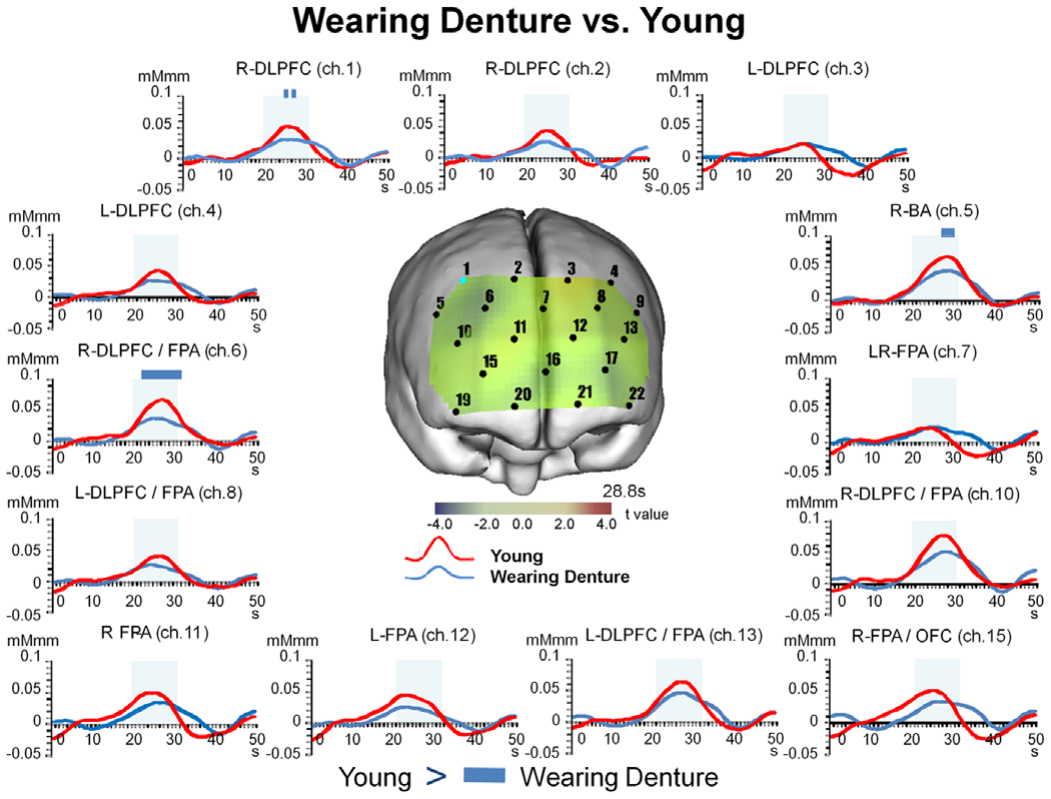
Temporal changes in [oxy-Hb] under Wearing Denture condition as compared to Young. The values for [oxy-Hb] under the Wearing were significantly (two-way repeated measures ANOVA and Bonferroni t-test, p<0.05) decreased as compared to the Young during the chewing period for DLPFC (BA9, BA46), FPA (BA10), and BA (BA45), and during the post-chewing period for DLPFC (BA46) and FPA (BA10). Significant differences between Wearing Denture and Young are indicated by a blue bar. All channels shown here correspond to channels that showed significant differences between the Wearing Denture and Tooth Loss presented in Fig 4.

When we compared the Wearing Denture condition with the Young, we found significant (Bonferroni t-test, p<0.05) decreases in the values for [oxy-Hb] during the chewing period for DLPFC (BA9, BA46, ch. 1, 6), FPA (BA10, ch. 6), and BA (BA45, ch. 5), and during the post-chewing period for DLPFC (BA46, ch. 6), FPA (BA10, ch. 6) (Fig 7 and Table 6-3).

## Discussion

The average masticatory score was 81.1 in the present partially edentulous elderly individuals and none had complaints associated with wearing a denture (Table 2). It has been reported that a score greater than 80 while wearing a denture indicates favorable chewing activity, which is correlated with chewing ability in regard to performance [39, 40]. Also, Aras et al. [74] reported an occlusal force value of 708.8±313.89 N (mean ± SD) during maximal jaw clenching while wearing a denture in subjects classified as B type in Eichner’s index, while Garrett et al. [75] reported a burst duration of 331.5±54.5 msec in subjects following adjustment of a poorly-fitting denture and when wearing a new denture, and Tallgren and Tryde [76] reported a peak amplitude of 0.16 mV in subjects with complete upper and partial lower dentures. Those results were similar to the present findings in regard to occlusal force (638.7±127.0 N) (Table 3), Mm EMG data for durst duration (327.9±75.2 msec), and peak amplitude (0.15±0.09 mV) (Table 4).

The present elderly subjects were classified as B2, B3, and B4 in Eichner’s index (Fig 1), of which B2 has 2 supporting zones, B3 has 1 supporting zone, and B4 has no supporting zones in the posterior part of the dental arch. Eichner’s index is strongly correlated to number of teeth [77] and occlusal force [78], and a previous multiple linear regression analysis showed that masticatory performance, determined by the concentration of dissolved glucose obtained from test gummy jellies, is significantly associated with posterior occlusal contact in Eichner’s groups B and C [78]. In addition, in groups A and B, the number of residual teeth was found to be significantly associated with masticatory performance, whereas it had no significant relationship with masticatory performance in group C [79]. Recently, Takeuchi et al. [80] reported that loss of posterior teeth occlusion was independently associated with cognitive decline, and that maintenance and restoration of posterior teeth occlusion may be a preventive factor against cognitive decline in aged individuals. We considered that it was reasonable to examine partially edentulous elderly patients rated B2-B4 by Eichner’s index to determine the association between chewing cognition and chewing activity in the present study. Individuals classified as B1 (unilateral edentulous) were excluded, as they are unable to chew gum bilaterally with a denture. Nevertheless, a greater number of cases is needed to more critically assess that association in all such subjects regardless of Eichner’s index classification.

We investigated dorsal prefrontal deactivation during the periods of preparation and execution of chewing performance under a tooth loss condition in partially edentulous elderly individuals (Fig 3A and Table 5-1), and then compared our results with those obtained while wearing a denture (Fig 4 and Table 6-1) as well as in young healthy control subjects (Fig 6 and Table 6-2). Our findings regarding modulatory masticatory muscle activity and decreased masticatory score caused by tooth loss may be associated with deactivation of the dorsolateral prefrontal cortex, which might induce cognitive decline by low masticatory performance under a tooth loss condition. Furthermore, we found increases in chewing activity by the masticatory muscles, masticatory score, and right hemispherical predominant prefrontal activity during the periods of chewing preparation and chewing execution under the wearing a denture condition (Figs 3B and 4, Tables 5-2 and 6-1). On the other hand, prefrontal activation induced by wearing a denture was not much different from that in the young healthy controls during chewing performance (Fig 7 and Table 6-3). Vermeij et al. [33, 81] presented results showing cognitive load-dependent activation of the prefrontal cortex in the same brain region in a healthy young control group, which may support the age-invariant view of compensatory prefrontal neural recruitment. From these findings, we consider that wearing a denture in the present partially edentulous elderly patients possibly compensated for recruitment of neural activities in the same prefrontal regions as those recruited in young healthy adults in response to increasing masticatory cognitive demand, as the results obtained under the condition of wearing a denture were not much different as compared to those in the young group. Nevertheless, additional study in regard to the age-invariant view of prefrontal compensatory neural recruitment during chewing performance is necessary to compare activation features in the prefrontal cortex and chewing behaviors between elderly dentate and young healthy adults.

Spraker et al. [82] and Derosière et al. [83] reported that a hand grasping task activated the right hemispherical dorsolateral prefrontal cortex for dynamic force generation, while Camus et al. [84] found that excitatory repetitive transcranial magnetic stimulation to the right hemispherical dorsolateral prefrontal cortex modulated food selection by healthy subjects. In addition, Chiang et al. [85] and Fleming et al. [86] noted that the junction of the right hemispherical dorsolateral prefrontal and frontopolar cortices was involved in metacognitive evaluation, as shown by masticatory score. Based on those reported findings, we speculated that right hemispheric dorsolateral prefrontal and frontopolar activation induced by wearing a denture may be responsible for force generation, food selection, and self-evaluation of chewing ability.

The prefrontal cortex is responsible for working memory, temporal processing, prospective coding, flexibility, and decision making, in addition to preparation and execution of movements [87]. Action execution principally requires modality-specific capacity and (re-)planning to engage modality-general working memory resources [88]. Furthermore, chewing performance may facilitate the activities of working memory and associated prefrontal activations [89, 90]. Moreover, since cognitive improvement was shown to be produced by extracranial excitatory repetitive transcranial magnetic stimulation [91–93] as well as transcranial direct current stimulation to the right hemispherical dorsolateral prefrontal cortex [94–96] in aged patients and those with Alzheimer’s disease, intrinsic masticatory activation induced by wearing a denture might also be useful for maintaining physiological cognitive ability in partially edentulous elderly individuals. Based on these findings, it is speculated that prefrontal activities in chewing preparation and execution under the condition of wearing a denture may reflect the capacity of not only chewing cognition, but also working memory in partially edentulous elderly individuals.

In summary, we found that tooth loss led to prefrontal deactivation accompanied by degradation of occlusal force, muscle activities and masticatory score, while wearing a denture improved prefrontal activation so that it was not much different from that in young healthy individuals in regard to chewing performance. Based on our results, we consider that wearing a denture may contribute to retain physiological chewing cognition in partially edentulous elderly individuals, whereas oral structural defects caused by tooth loss may collapse sensorimotor generation and integration within the masticatory system.

## Conclusion

In the present study, we found deficits in occlusal force, masticatory muscle activity, masticatory score, and prefrontal activation while chewing under a tooth loss condition as compared with wearing a denture and in young healthy controls. It is suggested that the chewing system may collapse from tooth loss in partially edentulous elderly individuals. In addition, our results showed that prefrontal activation induced by wearing a denture was not much different from that in young healthy subjects. We consider that improved chewing activities induced by wearing a denture may have physiological effects to retain chewing-related prefrontal cognition and associated cognitive functioning in partially edentulous elderly individuals. Finally, our findings suggest the presence of neurobiological markers to estimate cognitive activity in partially edentulous elderly subjects under the conditions of wearing a denture and tooth loss.

## Supporting Information

S1 Fig Dental state of 12 partially edentulous subjects. (XLSX)

S2 Fig Anatomical identification of near-infrared spectroscopy channels. (XLSX)

S3 Fig (A) Cross-sectional statistical topography under Tooth Loss conditions, as compared to Rest session (B) Cross-sectional statistical topography under Wearing Denture conditions, as compared to Rest session. (XLSX)

S4 Fig Temporal changes under Tooth Loss and Wearing Denture conditions. (XLSX)

S5 Fig Cross-sectional statistical topography in Young as compared to Rest session. (XLSX)

S6 Fig Temporal changes in [oxy-Hb] under Tooth Loss condition as compared to Young. (XLSX)

S7 Fig Temporal changes in [oxy-Hb] under Wearing Denture condition as compared to Young. (XLSX)

S1 Table Numbers of residual, lost, and prosthetic teeth in 12 elderly edentulous subjects. (XLSX)

S2 Table Masticatory scores for Tooth Loss, Wearing Denture, and Young. (XLSX)

S3 Table Occlusal force (N) for Tooth Loss, Wearing Denture, and Young. (XLSX)

S4 Table Masticatory muscle EMG activities for Tooth Loss, Wearing Denture, and Young. (XLSX)

S5 Table Significantly different channels and corresponding brain regions presented in cross-sectional statistical topography of [oxy-Hb]. (XLSX)

S6 Table Significantly different channels and corresponding brain regions presented in temporal changes of [oxy-Hb]. (XLSX)
